# Barriers to sustainable health research leadership in the Global South: Time for a Grand Bargain on localization of research leadership?

**DOI:** 10.1186/s12961-022-00910-6

**Published:** 2022-12-19

**Authors:** Marian Abouzeid, Ahlam Muthanna, Iman Nuwayhid, Fadi El-Jardali, Phil Connors, Rima R. Habib, Shahram Akbarzadeh, Samer Jabbour

**Affiliations:** 1 The Lancet-American University of Beirut Commission on Syria, Faculty of Health Sciences, American University of Beirut, Beirut, Lebanon; 2 Faculty of Health Sciences, American University of Beirut, Beirut, Lebanon; 3 Alfred Deakin Institute for Citizenship and Globalisation, Deakin University, Burwood, VIC Australia; 4 Centre for Humanitarian Leadership, School of Humanities and Social Sciences, Deakin University, Burwood, VIC Australia

**Keywords:** Leadership, Research leadership, Localization, Global South, Decolonization

## Abstract

**Background:**

Strong Global South (GS) health research leadership, itself both dependent on and a requisite for strong health research systems, is essential to generate locally relevant research and ensure that evidence is translated into policy and practice. Strong GS health research systems and leadership are important for health development and in turn for strong health systems. However, many GS countries struggle to produce research and to improve performance on widely used research metrics measuring productivity and reflecting leadership. Drawing on literature from a rapid review, this viewpoint paper considers the barriers to GS health research leadership and proposes strategies to address these challenges.

**Findings:**

GS researchers and institutions face numerous barriers that undermine health research leadership potential. Barriers internal to the GS include *researcher-level barriers* such as insufficient mentorship, limited financial incentives and time constraints. *Institutional barriers* include limited availability of resources, restrictive and poorly developed research infrastructures, weak collaboration and obstructive policies and procedures. *Structural barriers* include political will, politicization of research and political instability. External barriers relate to the nature and extent of Global North (GN) activities and systems and include allocation and distribution of funding and resources, characteristics and focus of GN-GS research collaborations, and publication and information dissemination challenges.

**Conclusions:**

Strengthening GS health research leadership requires acknowledgement of the many barriers, and adoption of mitigating measures by a range of actors at the institutional, national, regional and global levels. Particularly important are leadership capacity development integrating researcher, institutional and systems initiatives; new GN–GS partnership models emphasizing capacity exchange and shared leadership; supporting GS research communities to set, own and drive their research agendas; addressing biases against GS researchers; ensuring that GS institutions address their internal challenges; enhancing South–South collaborations; diversifying research funding flow to the GS; and learning from models that work. The time has come for a firm commitment to improving localization of research leadership, supported by adequate funding flow, to ensure strong and sustainable research systems and leadership in and from the GS. Just as the humanitarian donor and aid community adopted the Grand Bargain commitment to improve funding flow through local and national responders in times of crisis, we strongly urge the global health research community to adopt a Grand Bargain for research leadership.

## Introduction

The importance of research in improving population health and sustainable development is well recognized globally, as is the need for its localization—local actors empowered to use local contextual knowledge, lived experience and insights to identify and prioritize local issues, set the agenda, drive research and determine locally appropriate means of implementation. However, compared with those in high-income/Global North (GN) settings, many researchers and institutions in the Global South (GS) struggle to produce research and improve performance on widely used research metrics that measure productivity and reflect leadership.

Although some GS settings have shown slow progress [1], the GS is grossly underrepresented on journal editorial boards [2–4], volume of publication outputs [5–8], lead authorship on publications [9, 10] and health research funding [11]. Diseases with a high burden in the GN receive considerably more research attention than conditions only prevalent in low-income settings [12], and total investments in health research are disproportionately skewed away from the health issues particularly prevalent in and impacting the GS [13]. The GS is also underrepresented in leadership of research translation activities. A review of global public–private partnerships addressing neglected diseases highly prevalent in Africa reported that every such major organization examined was headquartered in the United States or Europe, none were led by a national of a GS country (most chief executive officers were male, all were Caucasian and all were residents of the United States or Europe), and only a very small proportion of executive director-level staff had non-United States or non-European origins [14].

## Defining and measuring (GS) health research leadership

Whilst conceptualizations and definitions abound, one definition of research leadership is “the degree to which the author (or country) assumes responsibility for directing the scientific work being developed” [9]. GS health research leadership can be considered as the capacity of GS researchers and institutions to advance values and skills required to generate local knowledge and solutions to local health problems and build a sufficient local cadre of skilled researchers [15]. Within a health research ecosystem, leadership exists at different levels, including individual, institutional, national, regional and global. There are also different types and parameters of research leadership, including publication leadership (based on publication counts); reference leadership (focusing on highly cited publications that have had considerable impact on the disciplinary field); and thought leadership (which captures ability to reflect and build on recent discoveries in the field) [16]. Various attributes and competencies of effective research leaders have also been described, and the personal traits and styles of successful research leaders may vary between cultures and contexts [17].

A range of indicators may therefore be used as proxy measures of leadership. Whilst there is no universally agreed or standardized set of metrics for health research leadership generally and GS health research leadership specifically, there are various readily available bibliographic and other measures, including country of affiliation of first, last and corresponding authors on research publications [5, 9], contribution of GS authors to various stages of the research process [18], patterns of national, intraregional, continental, interregional and GN–GS collaborations [9, 19–21], citation metrics by country of author affiliation or by collaboration patterns [9, 19], and a range of indicators used as part of research capacity development evaluation frameworks [22, 23], that may also reflect GS research and leadership capacity. Additional suggested indicators include ability to secure research income; ability to lead international research efforts; ability to support and deliver research training, including at the doctoral level; and ability to disseminate findings to inform national policy and practice [24].

## The importance of GS health research leadership

Strong GS health research leadership is a fundamental requisite for strong research systems and, by extension, strong health systems. Strengthening health research through a systems approach was the subject of a World Health Organization (WHO) initiative that commenced in the early 2000s and has included a key focus on strengthening systems in the GS [25]. This work has included describing the components and functions of health research systems, namely stewardship, securing financing, creating and sustaining resources, and producing and using research [26]—all of which require strong health research leadership.

Strong GS health research leadership is also integral to and interconnected with global commitments to improve health and development. For example, building institutional capacity and local leadership is at the core of the Sustainable Development Goals (SDGs); strong leadership of health research systems, which serve to build the evidence base and ensure translation of evidence into policy and practice and real-world impact, is fundamental to improving population health and particularly SDG 3. Strengthening GS leadership is also important for calls to decolonize global health [27], which have amplified and become increasingly pressing during the pandemic, given the gross power asymmetries in global health that COVID-19 has exposed [28].

## The stubborn inequities of health research leadership

Despite recognition of the importance of GS health research leadership, the reality remains that power imbalances and inequities continue to exist in research and in international collaborations, with GN countries, institutions and researchers often acknowledged as the research “leaders”, including through being assigned research leadership roles of first or corresponding author on collaborative publications [9]. This is reportedly sometimes the case even when GN collaborators do not have relevant local GS contextual knowledge. For example, in the Syrian conflict context, whilst much equitable and collaborative research has been conducted, GN–GS inequities in research collaboration have also been described, and these exist despite some international research partners reportedly being unfamiliar with the political economy, social context and nature of the conflict [29]. Compared with the prewar period, the Syrian conflict has also been associated with a decrease in the proportion of health-related publications on Syria that have at least one author affiliated with a Syrian institution [30]. Wars, armed conflicts and politico-economic instability can reverse progress achieved in health research leadership and associated health research metrics.

In recent decades, the global community has amplified efforts to bridge this GN–GS disparity, including by increasing health research funding and adopting new funding models to GS institutions and countries, supporting research education, implementing health research capacity-strengthening programmes, and supporting partnerships, networks and collaborations [31]. New networks and GS-led initiatives to address GN–GS disparities are also emerging, such as the Global Health Decolonization Movement in Africa [32]. Many such initiatives have had positive effects, increasing capacity and enabling some GS institutions and countries to improve research performance [33] and attain a pioneering research position compared with their neighbouring countries [34]. However, some such initiatives have also had the unintended effects of creating disparities in local knowledge generation within the same region and in GS–GS collaborations, leaving less developed countries further disadvantaged [34]. Despite gains in some GS countries, even many of those that have comparatively better research capacities remain, to varying extent, scientifically dependent and subordinate to their GN counterparts due to numerous structural impediments, including weak research leadership.

Strengthening GS health research systems and leadership and addressing GN–GS health research inequities require frank examination of barriers and identification of potential strategies to address them. Notably, there is no standard definition of the GS, but rather it is an umbrella term that has been ascribed a number of different meanings and boundaries which have also varied by discipline and over time; the classifications and definitions of the GS include using the term to denote poor, socioeconomically marginalized and/or (de)colonized countries, which themselves can be defined in various ways including through the World Bank’s Human Development Index and other such metrics [35]. Using this approach, the GS therefore entails a heterogeneous group of countries. There is marked variation in the political, economic, social, demographic and development indicators across GS countries, and this is also reflected in research and academic functions [8]. In some GS countries, there is limited supply and capacity of researchers and research institutes, whereas other countries have comparatively stronger research infrastructures and more expansive and active collaborative networks. For example, in a 2008 publication examining 10 developmentally diverse countries in the Eastern Mediterranean, considerable differences were documented in research productivity and in a range of national health research system characteristics, including national governance and management structures, national health research plans, priorities and policies, and research utilization [36]. Consequently, barriers and challenges to research leadership vary in different contexts; there is no “one size fits all” approach to health research leadership development.

## Barriers to GS health research leadership

In this viewpoint paper, we explore the barriers that hinder strong GS health research leadership, drawing largely on selected literature identified through a rapid search for English-language publications examining population health research leadership and published during the period January 2000 to March 2019. Notably, we do not consider leadership of biomedical, genomic or clinical research, research leadership in the GN, research leadership in other disciplines or leadership in any sector or organizational context other than research. Additionally, issues raised in documents published in languages other than English or in local or regional non-English publication outlets may not be captured. We draw on this literature to consider barriers internal and external to the GS and at the researcher, institutional and systems/structural levels and propose a set of recommendations for local, regional and global stakeholders to help address these issues and sustainably build GS research leadership.

### Barriers to leadership in and from the GS

Barriers internal to the GS exist at the researcher, institutional and systemic/structural levels (Table 1).

**Table 1:** Common themes of internal barriers to health research leadership in and from the GS

	Barriers
Researcher	*Faculty members* • Barriers to research productivity including lack of time, overloaded teaching schedule, lack of interest in conducting research, limited skills in research methodology and statistical techniques • Heavy teaching loads and administrative roles limiting the time for research • Not having defined workload allocation for teaching, research, administrative roles • Lack of incentives such as promotion to conduct research *Early-career researchers* • Limited mentorship opportunities • Poor research skills and capacity; basic research skills but lack of advanced research abilities • Limited research publishing capacity and publication challenges, including language barriers, time constraints, not knowing where to publish, prohibitive publication costs • Lack of incentives and motivation to publish, including lack of recognition through career development awards and low salaries • Limited grant-writing support and capacity, resulting in poor ability to attract international and local research funds *Research leaders* • Lack of recognition of the role of research in development or importance of operational research to programmatic activity • Weak research leadership skills and competencies
Institutional	*Personnel and institutional infrastructure* • Mainly teaching positions in higher education institutions, limited researcher positions • Shortage of faculty members and research leaders • Weak enabling environment: limited research assistants, limited funds for research • Poor research environment, weak health research system governance, infrastructure, policies and lack of institutional support • Corruption in research institutions *Research education* • Poor education in general, not limited only to research education • Weak research education/training in universities • Poor quality of doctoral research training • Limited funding to doctoral research training *Collaboration* • Culture of individualism • Limited collaboration between faculties or disciplines in the same university or institution • Weak collaboration between local research institutions • Limited collaboration and partnerships between programme staff and academic institutions, hindering operational research • Weak South–South collaboration intra- and interregionally *Knowledge translation* • Limited efforts from researchers and insufficient time, skills and institutional mechanisms for knowledge translation
Systemic/structural	*Political will* • Lack of political will to support research and poor local research funding *Politicization of research* • Limited freedom of research • Politicization of research, including research leadership roles • Political sensitivity of some research implications rendering knowledge translation challenging *Political instability* • Research infrastructures negatively affected by wars and conflicts • Loss of human capital: brain drain, push factors *Research systems* • Low number of health researchers, limited workforce capacity • Lack of research career pathways and limited funding for degree programme and postdoctoral research posts • Low researcher salaries • Much research undertaken through well-paid consultancies rather than through institutions, with commissioning bodies often unwilling to pay overheads to institutions • Poor recognition of research led by local researchers in GS countries themselves • Limited publication opportunities: few journals from the GS; poor indexing or low impact factors of local journals from the GS • Few and unsustainable research networks in GS or platforms to engage with regional and global research communities. Barriers to such networks include funding, weak and fragmented network management skills • Limited funding for capacity-building initiatives

*Researcher-level barriers* include those related to mentorship, language abilities, economic factors, including lack of financial incentives, and time. Difficulties with identifying appropriate local mentorship, and expectations that newly graduated researchers be immediately capable of securing independent funds, impact ability to retain emerging research leaders locally 37]. Time constraints and teaching and administrative functions consuming time that could be used for research are also described [38–40], with reports that faculty in some GS countries do not have clear workload allocations with a defined number of hours allocated for teaching, research and administrative roles [40]. Even after conducting and writing up good quality research, GS scholars may face publication challenges such as language barriers [39, 41, 42], time constraints [17, 40], and not knowing where to publish and prohibitive publication costs [43].

*Institutional barriers* include limited availability of resources such as funding [40, 44, 45], physical infrastructures [38, 44] and administrative support [38], limited incentives [17, 46, 47] and policies and procedures that can negatively impact research productivity. Institutions may also lack a sufficiently sized, trained and skilled research workforce [48] and some have only teaching career streams, with limited research job opportunities, research career pathways or allocated dedicated research time [49, 50]. Limited multidisciplinary and cross-sector collaborations and limited multi-institutional collaborations across the same country or inter-regionally [51, 52], can also prohibit GS leadership development. Limited leadership training opportunities are reported [17]. Poor research culture and / or unsupportive management have also been described [38, 44], including limited dissemination of information about available funding opportunities [11], limited access to professional development opportunities and publication assistance [42] and poor research culture due to unqualified research heads [44] and limited leadership in promoting research [45].

*Structural barriers* include political will, influence and instability. In some of the more politically stable GS countries, there is limited interest in research and limited awareness among policymakers of the importance of research in development, which is reflected in less political willingness to fund research [48, 53–58]. Authoritarianism and limited democracy can impede academic freedom; political power may also shape research leadership, as political favouritism may influence assignment of health research leadership positions or promotions in some GS countries [46, 59]. Political instability in fragile, conflict and violence-affected settings markedly undermine research leadership due to disruption of physical and information infrastructures, disruption of capacity development initiatives and training, security considerations, limited funding, and population displacement including academic brain drain [30, 45]. Weak national research infrastructures also generate challenges.

A range of factors also impact gender parity in research leadership [17].

### Barriers to leadership external to the GS

Barriers to GS leadership can also arise due to the nature and extent of activities and systems in the GN. These may be related to allocation and distribution of funding and resources; characteristics and focus of GN–GS collaborations; and publication and information dissemination challenges (Table 2).

**Table 2:** Common themes of external barriers to health research leadership in and from the GS

Factor	Barriers
Allocation and distribution of funding and resources	• Politics of research • Limited funds directed into research in lower-income countries • Limited impartiality in allocation of health research funding • Little funding and focus on building health research capacity in GS • Favouritism/factors other than merit influencing research commissioning
Characteristics and focus of GN–GS collaborations	• Limited focus on capacity-building of research leadership skills, e.g. partnership development skills, networking skills • Few GS leadership positions in GN-GS partnership projects, most leadership positions are held by GN research leaders • Power imbalance in social or economic capital and scientific capital (academic power) • Extractive and unequal relationships in partnerships • Unequal distribution of responsibilities in research stages with dominant and critical roles such as setting research plans and priorities, designing research questions and data collection tools, managing funds, data analysis undertaken by GN partners, while roles sometimes perceived as lesser such as data collection assigned to GS partners • Unequal distribution of lead authorship in research collaborations • Scientific imperialism in global health research • Colonial power in research capacity-building initiatives, paternalistic approach to building capacity • Support brain drain by acting as pull factors
Publication and information dissemination challenges	• Editorial board-related bias • Editorial independence and freedom potentially influenced by politics, journal owners, drug companies, political parties, mass media, scientists and researchers • Political sanctions may limit publication opportunities for researchers from those settings

#### Funding and resources

GS countries are generally heavily dependent on external funds to conduct research, given that GS governments commonly do not prioritize research and development and have limited funds on offer. However, even in GN-GS research partnerships, funding predominantly flows through the GN [60] and the fraction and absolute dollar value of research funding that flows to the GS remains low [12, 13, 61, 62]. Funding allocations and donor interest are sometimes ambiguous and not equally distributed amongst neighbouring countries with similar disease burden [63]. Rather, other factors such as historical colonial connections and economic and security interests may influence GN-GS research collaborations. Research-commissioning processes can also negatively impact GS research leadership [64, 65]. Research priorities are often determined by donors, resulting in research only addressing those issues for which funding is available and at the detriment of other priorities and national issues which remain under-researched [12, 62].

#### GN–GS Partnerships

The nature of GN–GS partnerships can impede GS research leadership. Gross power imbalances have been described, including regarding funding allocated to GS partners and allocation of roles and responsibilities, with GS partners often assuming or assigned to inferior and non-leadership roles, and made less visible and limiting ability to fully engage across activities [29, 48, 49, 60, 66, 67]: functions such as priority-setting and planning, formulating research questions and developing data collection tools, managing funds and conducting data analysis are often performed by GN partners, while GS partners generally conduct field work and data collection. Such inequities and power imbalances are also evident in publication authorship, with the first, last and corresponding authors often being authors with GN affiliations [5, 9].

In some instances, GN–GS partnerships are sometimes seen as unequal and extractive [39, 68] or even alienating and quasi-exploitative [67], allowing GN researchers to use the GS as a field or laboratory to gain research experience and enhance their research profiles or institutions more than that of the GS partners [69, 70]. Some partnership practices generate intellectual as well as financial dependencies. For example, in Zambia, there are reports that some GN collaborators build the capacity of Zambian partners to a level allowing them to better collaborate with GN partners but not for the essence of capacity-building of Zambian researchers [60].

#### Publication opportunities

Barriers to publishing hinder the visibility of GS research. Some GS journals have comparatively low international visibility, including due to not being indexed or having low citation scores [41, 43]. Editorial bias is described [39] and some journal editors are seemingly not interested in GS papers and do not recognize the challenges of conducting research in such settings [43]. Limited GS representation on editorial boards augments the publication barriers against GS-authored papers [4, 43]. Editorial decisions may be influenced implicitly or explicitly by various factors, including journal owners, drug companies, political parties, political sanctions against some countries, mass media, and other researchers [71].

## Given such barriers, how can GS health research leadership be sustainably strengthened?

### Leadership capacity development at the researcher, institutional and systems levels cannot occur in isolation

To address GS research leadership challenges, some have focused on developing capacity of researchers and networks rather than directly investing in building institutions [72]. Whilst upskilling individual researchers is important, it is neither a sufficient nor a sustainable approach—a well-trained researcher in an unsupportive environment will not thrive, and leadership gains and local investments are lost when a researcher leaves. Developing a sustainable health research system requires complementary capacity-building efforts at multiple levels, from the individual and institutional to the national and supranational [58, 73]. Several existing capacity development initiatives demonstrate the importance of concurrently addressing barriers at multiple levels, with impact varying with context. For example, the UNICEF/ UNDP/ World Bank / WHO Special Programme for Research and Training in Tropical Diseases (TDR) capacity-strengthening programme has delivered benefits to both researchers and institutions, with the support for institutional capacity development considered essential in the least developed settings where local investments into research were scarce and complementary to local efforts in more advanced GS settings [74].

Multipronged approaches addressing issues at multiple levels are required. These include institutional and systemic measures to address “brain drain”, through the creation of opportunities to train and gain research experience locally, providing incentives to attract and retain talent and encourage return of researchers who complete research training abroad, and availing mentorship opportunities [50]. For example, in the 1980s the Mexican Health Foundation funded the return of researchers, and developed health research prizes to stimulate national research [56]. Exploring mechanisms to engage diaspora researchers, the expertise and resources they bring and the mutual benefits of such connections also warrants consideration [49, 51, 75, 76].

Enabling infrastructures that (re)integrate researchers who complete training abroad is important. In Kenya, it is reported that skills acquired during doctoral training in the GN were not transferable to the GS because of limited facilities and technologies; pay discrepancies between GN and GS institutions also serve as a barrier to return [37]. Local mentorship is crucial for early-career researchers to support skill development and prevent dropout from GS research institutions to lucrative GN research bodies [37] or dropout from research altogether. The importance of mentorship is often overlooked and underestimated. In some settings, recent graduates are expected to immediately assume research leadership functions and expected to secure research funding [37] and drive independent research projects. Multinational mentorship programmes must ensure that mentors recognize the importance of context and the differences in availability of resources, conditions and access by setting. Further, leadership in mentoring is also necessary for institutional change [77]. Such mentorship requires cultural shifts: as stated by others, within institutions, GS research leaders should discard inferiority and superiority complexes and instead support personnel development and leadership at all stages [37]. Institutions must avail mentorship and development opportunities for early-career and seasoned researchers alike, thus developing and retaining a sustainable and skilled research workforce.

Like research, the global health education landscape is heavily skewed towards high-income countries, including in leadership and in course content and curricula focus [78]. The entire global health system needs to be revamped, and power asymmetries addressed, at individual and organizational levels [28]. Research and teaching go hand in hand. At the institutional level, the myth that developing strong teaching programmes should come before promoting research and innovations should be dispelled [79]. To be contemporary, teaching should be informed by research. Teaching improves if research grows. Institutions should adapt mechanisms, including researcher and institutional performance measures, that promote dual teaching–research functions. Notably, in some settings, research productivity reportedly declines with increasing seniority and when professor rank is attained and publications are no longer required for promotion purposes [38]. To remedy this, it has been suggested that incentives are required to encourage senior faculty to invest their time and experience into research [38, 40].

### New partnership models that encourage capacity exchange and shared leadership are required

There are various outdated and semi-colonial models of GN–GS research collaborations, such as “postal research” (data/samples collected by GS partners and sent to GN partners); “parachute research” (GN researchers fly in and out following a short visit for data collection); and “annexed site research” (research in the GS is managed by a GN team) [80]. These should be replaced by equitable models that work for all parties. For example, “partnership research”, based on a negotiated research agenda, local line management and developing local research infrastructure, has been proposed [80] and its operationalization and challenges in contexts such as South Africa reported [81]. Diaspora engagement and partnership is another potential model for GN–GS collaboration. Four principles underpinning truly cooperative partnerships have been described: mutual trust and shared decision-making; local ownership; ensuring research findings translate into policy and practice; and development of national research capacity [80]. Views of researchers from Uganda, Kenya and the United States on requisites for successful global health partnerships reported similar attributes: partnerships based on mutual respect and mutual benefit, trust, good communication and clear partner roles and expectations [82].

New approaches to partnerships and new mutually beneficial ways of working together that encourage bidirectional capacity development—that is, capacity exchange—are required. Small changes can have pronounced effects. For example, in allocating project roles, consideration of the potential benefits of particular tasks for collaborators is warranted. It is important that the GS contribution to developing capacity in GN researchers is also recognized. Whilst there is much emphasis on the GS benefiting from collaboration, skill transfer and the increased visibility of research that stems from collaborations with the GN, the GN also stands to benefit considerably from research in and with the GS, including, for example, gaining research experience in the GS context, testing an instrument or intervention in a new setting, or investigating a public health issue not present in the GN. When operationalized well, true GN–GS partnerships are a tremendous opportunity for bidirectional capacity exchange, with GN researchers also benefitting from valuable research training opportunities in GS settings.

Careful role allocation within partnerships is also important from a risk mitigation perspective. Unequal role allocations risk creating a dependency behaviour and weakening leadership among GS research leaders. If priorities are not aligned, assigning agenda-setting to GN partners without local input can steer research focus areas away from local GS population needs and priorities [66]. Collectively, these practices may attenuate the positive effects of GS research capacity development projects and risk damaging GS leadership, leaving GS researchers dependent not only on the economic capital of GN partners, but also on their scientific expertise.

### GS research communities must set, own and drive their research agendas

GS researchers have the capacity to set and drive their own research agenda, informed by local needs, local priority health issues and local information gaps. In reality, research agendas are often determined by donor funding priorities, which can also influence GS–GS collaborations, and the interests of GN partners. Such influences on priority-setting again reflect power imbalances and contribute to inequities.

New ways of priority-setting and commissioning research that empower GS leadership are essential, including mechanisms to support and encourage GS researchers to pursue new and potentially ground-breaking research, even on issues that are not necessarily focused on local community needs. Some have cautioned that individualistic, non-collaborative research activities not driven by community and national priorities and that only aim to expand the scientific profile or interests of a researcher or a research organization do not contribute to national capacity development [53]. However, just as in the GN researchers have the intellectual freedom to research and investigate novel ideas and issues, GS researchers must also have the opportunities and support to pursue new paths. Failing to do so risks creating missed opportunities for scientific breakthroughs and perpetuates the misperception that the GS is not equipped to make new discoveries or conduct novel research. Adopting a systems approach and national health research strategy that both encourages responsiveness to local need and promotes intellectual freedom, with associated funding streams that support both such approaches to priority-setting, is key.

### Biases and prejudices against the GS must be addressed

It is important for the global health research community and journal editors to recognize that tougher research environments, limited resources and political instability do not necessarily translate to poor-quality research from the GS, and to address any inherent biases and prejudices against GS research. In a blind study of 347 English clinicians, abstracts were more likely to be rated as being of high relevance and recommended to a peer when the authors’ affiliations were presented as being from a high-income country than when the source of the abstract was changed to suggest authors were from a low-income setting, and vice versa [83]. Study authors described this as unconscious bias and prejudice against GS authors. Others have described experiences of a journal editor rejecting a GS publication due to perceived lack of international readership interest in the local GS issue, and consideration by a potential GN collaborator that scientific research in a GS setting was not worth pursuing if it did not advance GN technological and intellectual developments [84]. Unless the global research community self-examines its own biases and addresses the structural and systemic barriers that implicitly or explicitly affect GS researchers and institutions, GS research will remain undervalued.

### GS institutions must address their own internal issues and drive cultural shifts

The GS must assume responsibility for its own internal structures and (dys)functions that serve to hinder strong and sustainable research leadership, many of which are often deeply embedded within broader societal constructs and cultures. For instance, in many settings (in both the GN and GS), corruption is widespread. The various ways in which corruption reportedly affects the university and research sector have been documented in a case study of Bolivia [85]; grant fiscal mismanagement in Africa, and the associated need for financial accountability, has been described [14]; appointments and promotions not based on merit have been reported [86]; and corruption in the broader global health sector, within both GN and GS settings, has been detailed [87]. At the researcher and institutional levels, cultural shifts and ensuring transparent accountability processes are required.

The GS is also responsible for creating local research-enabling environments—whilst GN–GS partnerships can support this, the cultural shifts, local collaborations and investments required are not the purview of GN partners. To create policy environments that draw on research, local policy-makers should value research in informing action. Limited receptivity of policy-makers to evidence and politicization of research and research findings are barriers that must be addressed. Engaging policy-makers in research and training policy stakeholders in basic research functions can facilitate this understanding of the importance of research. In Mexico, numerous such examples have led to increased policy-maker demand for and uptake of research and increased resources for research, with important benefits for local institution building [56]. Similarly, partnerships between programmatic staff and academic institutions may build capacity, provide a conducive environment and increase appreciation of the importance and relevance of operational research to programmatic activity [88].

### South–South collaborations should be facilitated and enhanced

Inequities within GS–GS collaborations can also be important, and collaboration within and between GS institutions is core to developing capacity and ensuring that those in the least developed contexts are not left behind. Exclusively developing capacity in selected GS countries and institutions risks amplifying regional inequities and further disadvantaging the most disadvantaged. For example, between 2005 and 2008, South Africa produced 78% of all intraregional co-authored papers, and 81% of all papers from within the 15-country Southern African Development Community. These findings led the author to conclude that South Africa has assumed a dominant role, similar to that assumed by the GN in GN–GS partnerships, of the scientific giant, with implications for brain drain due to South Africa’s ability to attract skilled personnel from less developed African countries, and incentivizing South African collaboration with the GN, from which it can benefit, above collaboration with other African partners [34]. Similar observations of some Arabic-speaking countries encouraging international rather than regional collaboration in order to increase research publication outputs are also reported [89]. From Egypt, across the broader health sciences literature published between 1980–2014, it is reported that more papers involved collaboration with local coauthors than with international coauthors. However, international collaborators were more likely to be from high-income and scientifically advanced settings than from elsewhere in the GS, and internationally coauthored papers had higher citation metrics than single-author or locally authored publications [90]. Strengthening promising GS institutions should ideally be linked to strengthening capacity in weaker GS institutions in the least developed contexts. Likewise, research capacity development initiatives such as GS doctoral and other research training programmes must ensure that South–South inequities are not exacerbated by selection processes and requirements that inadvertently disadvantage applicants from the least developed countries with poorer research track records or from non-English-speaking settings [74, 91].

### Diversified research funding flow to the GS must improve if visions for localization are to become a reality

GS researchers often need to compete for funding with GN researchers, who typically have stronger institutional support and business development infrastructures. Research funding, including through more investment by GS countries, can have a major impact on the ability of researchers and institutions to engage in research, attract and retain high-calibre staff, build research teams and capacity, and improve research production—which in turn impacts the ability to secure funding, and ultimately to build stronger and more sustainable institutions that are positioned to lead. GS governments and donors need to recognize the value of and invest in strong local research systems and follow through on commitments made. For example, in 2007 the African Union pledged to invest at least 1% of gross domestic product (GDP) in research and development, but this goal remains unrealized, with considerable variation across the continent [51]. Local investments can generate important gains. For example, Tunisia reformed its domestic research funding mechanisms, with more funding associated with a considerable increase in publication outputs [89]. GN donors also need to recognize that in a globalized world, strong GS research institutions and systems are important not only for the GS but also to GN interests. The COVID-19 pandemic has highlighted the importance of localization and locally led research and action, crucial for both local response efforts and global biosecurity in an interconnected world.

Donor practices can have differential impacts on GS researchers, institutions and systems. A diverse range of funding mechanisms and commissioning processes to support research capacity development, collaborations and partnership have been described in the East African context, with lessons and challenges noted with experiences from each [65]. Some donor practices can severely impact institutional capacity-building. A study of barriers to health-related social science capacity in East Africa reported that individually contracted research consultancies were common, with donors often reluctant to pay institutional overheads. Whilst these well-paid consultancies supplement salaries for researchers, they also divert staff from academic research and from training the next generation of researchers, restrict institutional research capacity and sharing of findings, and perpetuate a GN donor-driven research agenda [64]. Similarly, in a research partnership between a United States and Ugandan institution, insufficient funding of administrative and overhead costs for the GS institution due to United States fiscal administrative policies was said to drain rather than build capacity and undermine the African institution; programme partners suggested that in addition to research capacity development, addressing chronic underfunding and developing fiscal and administrative capacity are integral to equitable and sustainable GN–GS partnerships [68].

### There are many lessons to be learned from models that work

Whilst numerous barriers to GS research leadership have been documented, there are many success stories. For example, the Mentor–Protégé programme in Cameroon is helping mentor female researchers in order to address the gender gap in health research leadership [92]. The African Doctoral Dissertation Research Fellowship programme, a locally driven, multipronged regional research capacity-strengthening initiative, is developing research leadership at the researcher, institutional and regional levels, “producing and nurturing research leaders, strengthening university-wide systems for quality research training and productivity, and building a critical mass of highly trained African scholars and researchers” [91]. The University of Washington and Nairobi AIDS International Training and Research Program generated mutually beneficial training and collaborations between United States and Kenyan participants, and sharing of local and international resources which helped mitigate many of the local challenges of conducting research, supporting early-career investigators and eventually supporting transfer of research training capacity to a GS institution [93]. The Somali-Swedish Research Cooperation, reactivated following disruption during the civil war, has re-engaged three key partner groups (Somali universities, Swedish universities and Somali diaspora) in a successful Somali-owned collaborative capacity development effort that is based on equitable partnerships and a long-term cooperation vision [76]. The AuthorAID in the Eastern Mediterranean project has helped increase publication and dissemination of research results from the region through regionally led editorial mentoring [42].

Building on these lessons gleaned, we urge both GN and GS researchers and stakeholders to strive towards adaptive, contextually appropriate, adequately resourced, locally led initiatives that address local priorities, fill locally identified gaps and needs, and support local research leadership.

## Recommendations

Whilst this paper is focused on barriers to population health research leadership, many of the issues are relevant to and applicable across research disciplines. We encourage cross-disciplinary discussions among researchers and at the institutional, national, regional and global levels to identify common challenges and seek joint action. Building on the many suggestions from the literature, we propose a suite of recommendations targeting six sets of key stakeholders from both the GN and GS, engaged in the global health research arena (Box 1).

Box 1: Recommendations to promote GS health research leadership To governments, national research bodies and donors in the GS

- Establish country-specific models to generate resources to support national research
- Strengthen in-country capacity by increasing spending to support institutional research programme-building and postgraduate research training [94, 95]
- Promote the retention of researchers through creation of research career pathways, availing career development opportunities and providing competitive salaries for researchers [50, 96]
- Encourage return of researchers undertaking advanced research training in GN institutions
- Protect academic freedom
- Implement a reward system for publication [97] and for research translation activities
- Build political will and recognize the value of GS health research [58]
- Implement and support strengthening of a national health research system [62]

To research institutions in the GS

- Simultaneously drive research and teaching agendas
- Encourage diverse partnerships and networks between researchers and disciplines in the same university, nationally, regionally and internationally and implement a coordination framework to ensure sustainability [54]
- Create an enabling environment for conducting research and innovation, which may include providing necessary physical, human and financial resources
- Establish mentorship programs and standards for mentorship and scientific development [11], and provide sufficient and sustainable research training and mentoring opportunities [93]
- Incorporate research leadership capacity development into institutional strategic plans
- Identify and address barriers related to institutional policies, procedures or cultures
- Cultivate both research leadership and leadership in research management [17, 58]

To global health and research governance partners

- Establish indicators to monitor GS research leadership, including capacity and funding flow to GS investigators and institutions.
- Consider drafting a “Grand Bargain” for research leadership and encourage signatories to commit to strengthening research localization.
- Promote equitable partnership models and culturally sensitive guidelines for GN–GS collaboration.

To research funders

- Fund and create more doctoral and postdoctoral research education opportunities in the GS for promising researchers from the GS
- Earmark specific funding to GS countries for research leadership capacity development activities and track the impact of such funding [98]
- Ensure sufficient funding of overhead costs for GS partner institutions, and incorporate support for fiscal and administrative capacity-building [68] and to support physical infrastructures [96]
- Encourage and incentivize equitable GS–GS partnerships and networks [50, 96]
- Coordinate with other donors [95]

To research institutions in the GN

- Ensure that GS health research leadership capacity strengthening, with priorities defined locally, is a priori an integral component of any international research collaboration involving GS researchers and institutions
- Recognize the value of capacity exchange, and respect the knowledge and expertise of GS partners
- Commit to equitable roles and responsibilities among GN and GS partners in international collaborative research projects
- Consider the role that diaspora researchers may play in helping bridge the GN–GS divide

 To international/global health journals

- Improve GS representation on editorial boards towards a 50–50 GN–GS balance
- Establish regional editorial offices in the GS [43] which are run in partnership with local institutions in order to ensure sustainability
- Actively commission work from GS countries [43]
- Devote space regularly to promote GS health research leadership
- Waive publication charges for authors from the GS, particularly low- and low-middle income countries [99]
- Provide editorial support to researchers who are not native English speakers
- Support editors of GS journals to improve quality of publications and increase visibility of research from the GS

Strengthening national research leadership is a local process and must be driven locally. No matter how much global support exists, efforts to strengthen health research leadership would falter without local ownership, nurturing and funding. Global actors must work in concert with local ones to address the challenges facing GS health research leadership, including across areas such as global research funding flows, research prioritization, research conduct and research governance. To ensure accountability of efforts to increase GS health research leadership, a standardized set of metrics are required to track progress, similar to proposed approaches for measuring and tracking localization in the context of humanitarian aid delivery [100]. Such metrics should capture gaps and needs, funding flows, initiatives and GS health research leadership progress. A standardized set of metrics would overcome the twin challenge of multiple definitions and dimensions of health research leadership and the lack of uniform indicators that consistently and comprehensively assess research leadership capacity and performance in and between GS countries, and indeed between the GN and GS.

Finally, just as the humanitarian donor and aid community adopted the Grand Bargain, a commitment to improve funding flow through local and national responders in times of crisis, we strongly urge the global health research community to adopt a Grand Bargain for research leadership. The time has come for a firm commitment to improving localization of research, supported by adequate funding flow, in order to ensure strong and sustainable research systems and leadership in and from the GS. This way the GS is sufficiently equipped and resourced to research, identify and respond to its own health issues. While recognizing the immense value of GN–GS collaborations, GS leadership must be sustainably strengthened, and the GS must lead the way on research in and on GS issues—nothing about us, without us.
